# Mpox: current knowledge and understanding—a scoping review

**DOI:** 10.1093/femsre/fuaf025

**Published:** 2025-06-09

**Authors:** Helen Callaby, Amy Belfield, Ashley D Otter, Barry Atkinson, Michael Reynolds, Helen Roberts, N Claire Gordon

**Affiliations:** Institute of Medical Sciences, University of Aberdeen, Aberdeen, AB25 2ZD, United Kingdom; UK Health Security Agency, Rare and Imported Pathogens Laboratory, Porton Down, SP4 0JG, United Kingdom; Emerging Pathogen Serology Group, Vaccine Development Evaluation and Preparedness Centre (VDEP), UK Health Security Agency, Porton Down, SP4 0JG, United Kingdom; Diagnostics and Pathogen Characterisation, UK Health Security Agency, Porton Down, SP4 0JG, United Kingdom; Emerging Infections and Zoonosis, UK Health Security Agency, Colindale, NW9 5EQ, United Kingdom; Emerging Infections and Zoonosis, UK Health Security Agency, Colindale, NW9 5EQ, United Kingdom; UK Health Security Agency, Rare and Imported Pathogens Laboratory, Porton Down, SP4 0JG, United Kingdom

**Keywords:** mpox, transmission, vaccination, infection control, clade I, clade II

## Abstract

Mpox in humans is a rash illness resulting from infection with monkeypox virus (MPXV). In 2022, a public health emergency of international concern (PHEIC) was declared with 115 countries reporting cases of Mpox. Most of these countries had not previously reported cases. This global outbreak was sustained primarily by human-to-human transmission within complex sexual networks. Whilst these cases were similar to previous clade II West African MPXV isolates, they were sufficiently genomically distinct to result in WHO recognizing two subclades within clade II: clade IIa and clade IIb. In 2024, a second PHEIC was declared, resulting from a marked increase in cases of clade I MPXV. In this scoping review, we compare the major clinical, epidemiological, and genomic features of the major mpox lineages and the implications for vaccination, transmission, infection control and treatment..

## Teaser

A scoping review, comparing the major clinical, epidemiological, and genomic features of clades I, IIa, and IIb MPXV.

## Introduction

Mpox in humans is a rash illness resulting from infection with monkeypox virus (MPXV). Whilst the clinical syndrome is similar to smallpox, it is typically less severe. In addition, MPXV is maintained by animal reservoirs meaning that in contrast with smallpox, mpox eradication (the status whereby no further cases occur anywhere) is unlikely to be achievable (Gispen 1975, World Health Organization (WHO) 1980). Elimination (the disappearance of a disease from an area) may however be possible, and some countries, including the UK, are directing their efforts at eliminating mpox from the population whilst recognizing that imported cases may still occur.

MPXV was first identified in 1958 following recognition of pox-like disease in laboratory primates imported into Copenhagen (Magnus et al. 1959); however, it was not until over a decade later that it was recognized as a potential human pathogen.

The first case of human mpox was identified in the Democratic Republic of Congo (DRC) in 1970, in a 9-month-old child who presented with fever followed by a rash 2 days later (Ladnyj et al. 1972). The rash was centrifugal and similar to smallpox, but unlike smallpox, it was accompanied by lymphadenopathy. Between 1970 and 2017, human cases and occasional outbreaks were reported in countries in Central and West Africa, usually associated with zoonotic transmissions (Heymann et al. 1998, Durski et al. 2018). An outbreak in the USA in 2003 was traced to importation of infected rodents from Ghana that were housed with prairie dogs subsequently sold as pets (Centre for Disease Control and Prevention (CDC) 2003). In 2017, after an apparent absence of over a decade, a cluster of human cases was identified in Nigeria, although it is likely cases were occurring but remained officially unidentified (Rimoin et al. 2010) or unreported. Since 2017, Nigeria has reported ongoing human cases associated with waning smallpox immunity and increased human-to-human transmission (Precious et al. 2023).

MPXV has two recognized major lineages: clade I, formerly known as the Central African or Congo Basin clade, and clade II, formerly known as the West African clade.

In 2022, a public health emergency of international concern (PHEIC) was declared with 115 countries reporting cases. Most of these countries had not previously reported cases. This global outbreak was sustained primarily by human-to-human transmission within complex sexual networks (Diaz Brochero et al. 2025). Whilst it was apparent that the genomic sequences of viruses associated with these global cases were similar to previous clade II West African MPXV isolates, they were sufficiently genomically distinct to result in WHO recognizing two subclades within clade II: clade IIa, containing the strains previously identified in West Africa, and clade IIb, containing the majority of strains associated with the global epidemic (World Health Organization (WHO) 2022).

In 2024, a second PHEIC was declared, resulting from a marked increase in cases of clade I MPXV. As with the 2022 PHEIC, this increase was linked with sustained human-to-human transmission, and genomic analysis showed distinct differences compared to historic clade I sequences (Vakaniaki et al. 2024). This resulted in the clade I lineage being further divided into clades Ia and Ib, with the overwhelming majority of cases identified outside of endemic countries being caused by clade Ib MPXV. Concurrent with the human transmission chains, some of the identified 2024 clade I cases in the African region were caused by zoonotic introduction (Kinganda-Lusamaki et al. 2025a).

In this scoping review, we compare the major clinical, epidemiological, and genomic features of the major mpox lineages and the implications for vaccination, transmission, and infection control.

## Virology and virulence

MPXV is a double-stranded DNA virus classified within the genus *Orthopoxvirus*. The viral genome is ~200 kb, coding for 181 proteins. The central section of the genome is conserved between orthopox viruses, whilst the terminal regions (33 225 and 62 157 bp, respectively) vary between species and encode the proteins thought to be responsible for differences in host range and pathogenicity (Karagoz et al. 2023). For MPXV, clades I and II differ in insertions and deletions in the inverted terminal repeat regions, which may explain differences in virulence between the two clades (McCollum and Damon 2014).

## Clinical signs

Clades I and II MPXV strains also appear to differ clinically. Clade I is typically associated with increased severity, including widespread rash, higher hospitalization, and a higher case fatality rate (Likos et al. 2005).

Based on the initial cases, mpox was considered a systemic infection, presenting like smallpox with a febrile prodrome during the initial viral replication stage, followed by a widespread rash (Mandell, Douglas and Bennett’s Principles and Practice of Infectious Diseases 2015). Historically, the mpox rash was described as progressing from papular to vesicular to pustules before crusting and desquamating, with all lesions progressing through the stages at the same time, similar to the smallpox rash. Unlike smallpox, the mpox rash was accompanied by lymphadenopathy in most patients, sometimes involving only regional lymph nodes but often more generalized (Mandell, Douglas and Bennett’s Principles and Practice of Infectious Diseases 2015). No equivalent of the flat-type or haemorrhagic-type variola rash has been described for mpox (Fenner et al. 1988).

Over the past few years, it has become apparent that mpox is a more diverse illness, with atypical rashes and the recognition of cases where only mucosal disease is present. The different presentations appear to be somewhat related to clade, with transmission route and host factors also playing a potential role (Bunge et al. 2022).

Historically, in clade I MPXV outbreaks, patients commonly have a febrile prodrome, as noted in over 99% of cases in one outbreak (Ogoina et al. 2023). The rash is often monomorphic and the distribution centrifugal with the primary rash site often being the face (82%) (Whitehouse et al. 2021). Genital rash was previously only reported in a quarter of cases. 80% of patients had lymphadenopathy (Ogoina et al. 2023). 75% of cases between 1981 and 1986 had >100 lesions and were critically unwell (Mandell, Douglas and Bennett’s Principles and Practice of Infectious Diseases 2015). The CFR was estimated to be ~10.6% (Hoffmann 2024).

In the current clade Ib outbreak in the DRC, one study of 226 suspected cases in South Kivu demonstrated that all patients had rash, over half had fever (59%), and 40% had lymphadenopathy (Vakaniaki et al. 2024, Masirika et al. 2024b). Non-specific symptoms such as headache and myalgia each affected ~1/5 patients (Katoto et al. 2024). Of note, transmission appears to include sexual contact, and symptoms also include genital lesions (Kibungu et al. 2024; World Health Organization (WHO) 2024a; Brosius et al. 2025). A prospective observational cohort study in South Kivu found that 88% (331/375) of patients had a prodromal phase to their illness, and 98% (396/404) of patients had a rash with a median lesion count of 42 at admission (Brosius et al. 2025). The rash was generalized in 35% of cases (137/394). Only 5% of patients had a documented fever (>38°C) at the time of inclusion in the study. 73% had lymphadenopathy (288/394) and this was more common in those >15 years (82%) compared with those <5 years (19%). In Burundi around half of the patients were children under 15 years old, which differs from the DRC (Nzoyikorera et al. 2024). A study of patients seen in a Bujumbura hospital in Burundi found that rash was reported in nearly all patients for whom data was available (249/250): pustular/vesiculopustular rash was most common (92%) with generalized rash in over three quarters of patients (78.3%) (Nizigiyimana et al. 2024). Genital rash was only reported in 19.7% of patients. Half of the patients had a fever (50.4%), and over one-third had lymphadenopathy (36.8%) (Nizigiyimana et al. 2024). Headache was reported in 19.6% of patients. No deaths were noted, although 45 patients were still hospitalized at the end of the study period (Nizigiyimana et al. 2024). A small study of eight pregnant women in South Kivu described miscarriage or stillbirth in 50% (Katoto et al. 2024, Schwartz 2024). The current CFR in Africa is estimated to be 2.73%; however, this is noted to be higher in provinces with more paediatric infections such as Equateur, DRC (Africa CDC 2024; McQuiston et al. 2024). Notably, there is widespread variation in the reporting of lymphadenopathy, which, given that it is an examination finding subject to clinician variability, may not be altogether surprising.

For the clade IIa outbreak in the USA in 2003, data available from 35 patients showed that 85% had a febrile prodrome or fever accompanying the rash (Centre for Disease Control and Prevention (CDC) 2003). The rash typically progressed through the classic stages, although in some patients early lesions did ulcerate (Centre for Disease Control and Prevention (CDC) 2003). The rash was varied with lesions occurring on the head, trunk, arms, and legs, including palms and soles, and over 77% of patients had respiratory symptoms (Centre for Disease Control and Prevention (CDC) 2003). 69% of the patients had lymphadenopathy (69%). There were no fatalities (Centre for Disease Control and Prevention (CDC) 2003).

In 2017–2018 an outbreak of clade IIb occurred in Nigeria (Ndodo et al. 2023), with 122 confirmed/probable cases who all had a vesiculopustular rash (Yinka-Ogunleye et al. 2019). Rash mostly affected the face, legs, and trunk (96%, 91%, and 80%, respectively). Genital rash was seen in 68% of patients. There was a range of distributions of rash, and over half were monomorphic. The majority also had a fever (88%) and lymphadenopathy (69%). Non-specific symptoms included pruritis, myalgia and sore throat, all of which were seen in over 50% of patients. During 2018–2021 there were seven patients diagnosed with mpox in the UK (Adler et al. 2022). Three were separate index cases with travel to Nigeria; the others were transmissions from the index case. All patients had a pleomorphic rash, and 5/7 (71%) had genital lesions (Adler et al. 2022). 4/7 had a prodrome (57%), including fever, headache, groin swelling and coryzal illness. 5/7 (71%) had lymphadenopathy.

During the global outbreak in 2022, most cases were due to clade IIb MPXV (Laurenson-Schafer et al. 2023). Compared to previous outbreaks, symptoms were less severe and CFR was <1% (Laurenson-Schafer et al. 2023). WHO data indicates that the majority of patients reported a rash (80%), including genital rash in 46.6%. Of note, genital rash was noted to be more common in males >15 years old (47.1%) than females >15 years old (27%). Over half of patients had a fever (59.6%), and lymphadenopathy was noted in 30%. Non-specific symptoms included headache, myalgia, and fatigue. Pauci-lesional disease was frequently reported: one large case series showed that most patients had 10 lesions or fewer, lesions were often pleomorphic rather than showing the classic vesiculo-pustular progression, and the anogenital area was the most commonly affected anatomical site (Thornhill et al. 2022).

Complications of mpox also vary with clade as shown in Table 1.

**Table 1.** Complications of mpox by clade.

Clade	Complications	% with complications	Case fatality rate
Ia	Skin infections Respiratory disorder Gastrointestinal disorder Keratitis/ocular disease Pregnancy complications (Mbala et al. 2017, Thornhill et al. 2022)	40% (Nizigiyimana et al. 2024)	5%–17% (Breman et al. 1980, Jezek et al. 1987) (note that early evidence from PALM007 trial indicates much lower mortality (1.7% with optimal supportive treatment) (National Institutes of Health 2024)
Ib	Surveillance ongoing Neuro-ophthalmic complications, e.g. meningitis/encephalitis, conjunctivitis/uveitis Loss of vision (unclear if transient or permanent) Pregnancy complications Genito-urinary complications, e.g. urethritis, genital oedema Cutaneous complications, e.g superadded infection and abscess Scarring (Mandell, Douglas and Bennett’s Principles and Practice of Infectious Diseases 2015)	Surveillance ongoing (66% in one study)	(Huhn et al. 2005, Ogoina et al. 2023, Desai et al. 2024, Vakaniaki et al. 2024, Masirika et al. 2024a)
IIa	Keratitis/ocular disease Encephalopathy Respiratory lymphadenitis with dysphagia and airway compromise	8.1%	0%
IIb	Proctitis/Anorectal pain Superadded infection Abscess Pharyngitis Ocular disease AKI Myocarditis Low mood (Adler et al. 2022)	10.8%	3%–5% for lineage A (data from Nigerian studies, mostly people with advanced HIV); (Yinka-Ogunleye et al. 2019, Ogoina et al. 2020, Ogoina et al. 2023) 0.19% for lineage B.1 (mostly people with advanced HIV) (Thornhill et al. 2022, Laurenson-Schafer et al. 2023)

In summary, skin manifestations, particularly rash, are a predominant feature for all patients with mpox infection. Historically clade I rashes have been monomorphic; however, the nature of the rash is unclear in the current clade Ib outbreak. Clade II rashes are typically pleomorphic. Genital rash has been reported in most clades but is more commonly reported in clade IIb, which may reflect the primary inoculation site associated with the predominant mode of transmission for this clade.

Most patients with clade Ia have a fever and lymphadenopathy, whilst both symptoms are present but less common in clade Ib and clade II. Currently, rates of complications and CFR are significantly higher in clade Ia than in the other clades, although it should be noted that much of the clade I data come from low-income settings where patients with mild disease may not present to healthcare services and therefore are not represented in the figures, and where there may be limited access to supportive treatment for the more severe cases, resulting in higher rates of complications, secondary infections, and fatalities.

## Zoonotic risk

MPXV has a wide range of animal hosts, allowing it to maintain a reservoir in wild animals. Historically, sporadic human cases have been associated with close animal contact such as hunting and skinning for bushmeat in endemic countries (WHO 2024), with occasional single human cases exported to non-endemic countries. In 2003, a large outbreak of clade IIa MPXV in humans in the USA was associated with the importation of infected rodents from West Africa. Some of the rodents were temporarily co-housed with native prairie dogs in an animal facility; the prairie dogs were then sold as pets prior to developing signs of infection and subsequently transmitted the virus to their owners. In total, there were 47 confirmed and probable human cases with no deaths and no human-to-human transmission reported (Reynolds et al. 2006).

Evidence suggests that rodents such as giant pouched rats, chinchillas, squirrels, marmots, and prairie dogs are susceptible to infection and may act as reservoirs (Reynolds et al. 2019, Centre for Disease Control and Prevention (CDC) 2024), and, as demonstrated by the 2003 US outbreak, imported animals may be a potential source of infection, either directly or by infecting co-housed animals. In the EU, there are no harmonized health certificates for the commercial importing of rodents from third countries, and MPXV is not notifiable: Importations of animals intended for the exotic pet trade are therefore possible and may initially go unnoticed.

Pox viruses, including MPXV, are stable in the environment and can persist for prolonged periods. Exposure of wild or pet rodents to contaminated household rubbish is a potential route for reverse zoonotic transmission. This should be considered when human cases are identified. Currently, there is no evidence to suggest differences between clade I and clade II in terms of animal susceptibility.

## Transmission

Attempts to identify the routes of transmission for mpox cases have been made since MPXV was first identified as a human pathogen with numerous analyses performed (Bunge et al. 2022). The first large-scale study of mpox cases in endemic regions (*n* = 338) performed in the early 1980s identified that 72% of mpox cases related to primary zoonotic spillover events following contact with wild animals and 28% of cases developing mpox following contact with another human case (Jezek et al. 1988). Notably, only 23/338 (6.8%) of cases were 15 years of age or older (Jezek et al. 1988). Investigations into a large outbreak in 1996–1997 (*n* = 419) also observed a high attack rate in children (85%); however, this study identified 22% as being primary cases and 78% as secondary cases (Centers for Disease Control and Prevention (CDC) 1997).

Several possible explanations for the preponderance of cases in children have been proposed, including increased contact with zoonotic reservoir species and less developed immune systems; however, the impact of immunity conferred from the global smallpox eradication programme is likely an important driver. The smallpox vaccine offers ∼85% protection against development of clinical mpox disease (Fine et al. 1988); however, the cessation of smallpox eradication programme in 1980 resulted in the majority of the population lacking vaccine-derived immunity from the turn of the millennium when a notable increase in mpox cases was recorded in endemic regions (Rimoin et al. 2010).

Although the route of transmission for primary cases involves direct contact with known or potential animal reservoirs of MPXV, less is known about the routes of transmission resulting in secondary cases. It is likely that direct contact (which may be sexual direct contact or non-sexual direct contact) is the predominant driver of human-to-human transmission; however, the contribution of fomite transmission and respiratory transmission cannot be excluded. It has been demonstrated in both healthcare and non-healthcare settings that significant contamination of the environment occurs around confirmed cases, including recovery of infection-competent MPXV (Atkinson et al. 2022, 2023, Gould et al. 2022). Whilst this implies potential for human-to-human transmission via fomites, most people within these contaminated environments will also have potential for direct contact transmission. A study of 1526 household contacts of mpox cases in the UK found a secondary attack rate of 4% (60/1526) with an extremely low risk of infection in non-sexual household contacts despite the potential for significant non-sexual direct contact in these settings (Packer et al. 2024).

The potential for respiratory transmission is even more enigmatic due to complexity of excluding other potential routes of transmission. Whilst mpox infection via the respiratory route has been demonstrated in several animal models (Beeson et al. 2023), these studies frequently use high inoculum titres and other experimental factors that are unlikely to replicate respiratory infection in humans. The strongest evidence for this route of transmission in humans to date relates to a single case in a healthcare worker in the UK who is believed to have been infected after changing the bed linen of an mpox patient prior to their diagnosis (Vaughan et al. 2020). The potential for MPXV to be aerosolized via a bed linen change was further supported when MPXV was isolated from an air sample collected during a bed linen change for an mpox patient in 2022, thereby demonstrating that infection-competent MPXV can be present in the air following this activity (Gould et al. 2022).

Epidemiological data imply that minimal respiratory transmission occurs with MPXV due to the absence of cases with only close proximity to infected patients but no direct contact; however, it is possible that transmission via the respiratory route may occur in specific instances especially with aerosol-generating techniques.

Data relating to the potential and propensity of vertical mpox transmission are sparse. Vertical transmission resulting in foetal demise has been demonstrated in an animal model with MPXV DNA identified in foetal tissues from two infected pregnant macaques; importantly, a third infected macaque was euthanized following signs of impending miscarriage with no MPXV DNA in foetal tissue inferring mpox infection can result in negative pregnancy outcomes without infecting the foetus (Krabbe et al. 2024).

Human cases of mpox during pregnancy are infrequent; however, several cases with negative foetal outcomes have been reported albeit few with assessment of foetal tissue to demonstrate vertical transmission. At least 32 cases of mpox during pregnancy have been reported in the scientific literature with 50% of cases reporting gestational outcome (6/12) resulting in foetal demise (Sanchez Clemente et al. 2024). Similar complexities exist with the potential for postnatal vertical transmission via breastfeeding. Whilst at least one case has been reported, it is likely transmission occurred from direct skin-to-skin contact rather than via infected breast milk (Alonso‐Cadenas et al. 2023). It is likely that vertical transmission of MPXV can occur during pregnancy; however, even if the likelihood of transmission is low, other negative outcomes may occur without transmission of the virus to foetal tissue. As a result, cases of mpox during pregnancy are a significant concern requiring specialist medical attention to support both mother and foetus when cases are detected.

It may be assumed that the overt nature of many clinical mpox cases would help mitigate the potential of onward transmission especially when sexual contact is the main driver for an outbreak or epidemic; however, the propensity for asymptomatic and pre-symptomatic mpox transmission increases the potential for onward transmission when outbreaks occur. Modelling based on transmission pairs in a cohort of 2746 mpox cases in the UK suggests that there may be considerable pre-symptomatic transmission, and that symptom development in the primary case may occur up to 9 days after the transmission event (Ward et al. 2022). Asymptomatic mpox has also been reported in many countries with up to 6.5% of people not experiencing indicative symptoms (De Baetselier et al. 2022, Ferré et al. 2022, Agustí et al. 2023). Collectively, the potentially high rates of pre-symptomatic transmission make public health responses to mpox outbreaks challenging and indicate that some transmission events may occur without overt symptoms in infected individuals.

The presence of two phenotypically different clades of MPXV raises the potential for alterations in transmission potential and propensity between clade I MPXV and clade II MPXV, in addition to differences between the sublineages of each clade. At present, there are not enough data to elucidate whether such differences occur. Further studies are needed to improve our understanding of MPXV transmission for all clades and sublineages. Many such studies can be performed *ex vivo* such as studying survival on surfaces and aerostability; however, epidemiological studies during outbreaks may offer significant insight into how MPXV transmits between individuals especially if such studies can identify cases from non-contact modes of transmission.

The 2022 clade IIb PHEIC demonstrated genomic evidence of human immune system viral editing via APOBEC3 deaminase inferring prolonged human transmission chains not previously witnessed prior to this global epidemic (O'Toole et al. 2023). These molecular signatures confirmed the broader epidemiological findings of the PHEIC, namely that mpox was no longer primarily a zoonotic disease with limited human-to-human transmission, and now was a disease capable of establishing prolonged transmission chains in humans. Whether APOBEC3 deaminase editing is the cause or effect of this epidemiological paradigm shift is unclear; however, the future of MPXV transmission will likely involve sustained human-to-human transmission chains with ABOPEC3 signatures in viral sequences. Analysis of available sequences relating to the 2024 clade Ib PHEIC also showed evidence of APOBEC3 deaminase editing (Vakaniaki et al. 2024, Kinganda-Lusamaki et al. 2025b) and may explain why this outbreak has persisted longer than any previous clade I MPXV outbreak. It is tempting to speculate whether APOBEC-related viral editing will further impact future MPXV transmission, in particular the potential to select for less overt clinical manifestations or longer prodromal phases, but more information is needed before a conclusion can be reached. Until then, public health responses need to mitigate the potential for onward transmission through clear messaging to at risk populations and scientific research must try to disentangle some of the enigmatic aspects of potential MPXV transmission particularly in relation to potential fomite and respiratory transmission.

## Infection control

A 2024 systematic review aimed to synthesize all evidence of infection and prevention control measures to mitigate mpox transmission, yet their search terms yielded no results in this area, demonstrating the current lack of research and poor evidence base for control measures (Kuehn et al. 2024). The study authors concluded that based on what is published to date, there is little evidence of benefit from the use of a respirator mask (FFP3) versus a fluid-resistant surgical mask and that there is equally unlikely to be a difference in transmission from the use of an airborne precaution ventilated isolation room versus an adequately ventilated single room (Kuehn et al. 2024).

However, as outlined above, pre-symptomatic and asymptomatic transmission have described (Ward et al. 2022), indicating that routes other than skin contact remain important, and a precautionary approach is generally adopted (58) when assessing and caring for mpox patients in the hospital setting because of a lack of available data.

Current guidance from the World Health Organization (WHO) is similarly cautious, and recommends concurrent contact, droplet, and respirator precautions for MPXV (World Health Organization (WHO) 2022). WHO acknowledge the limited evidence for continuous use of airborne precautions, but given the uncertainty, continue to recommend the use of a respirator mask whilst advocating for further research (World Health Organization (WHO) 2022).

WHO also acknowledge that it may not be possible to follow this guidance in resource-limited settings, particularly for home isolation, and have produced separate guidance accounting for this (World Health Organization (WHO) 2024b).

## Diagnostics

Primary diagnostic testing is usually by real-time Polymerase Chain Reaction (PCR), performed on a lesion swab or aspirate. The G2R_G gene is used to differentiate MPXV from other orthopoxviruses (Li et al. 2006). Several commercial real-time PCR kits for detection of OPXV and/or MPXV are now available (Michel et al. 2022, Papadakis et al. 2023).

Previously, clades I and II were differentiated by the presence/absence of the C3 L gene, which is located in the more variable terminal region of the MPXV genome. The G2R-WA target is conserved in all clade II strains but is present to a variable degree in clade I, and until recently, a combination of C3 L and G2R-WA has been the approach of choice for determining whether a strain belonged to clade I or II (Li et al. 2010).

However, the emergence of the Ib sublineage in the DRC in 2023/2024 has required further assay development. The Ib sublineage lacks the target region in the C3 L gene, which may therefore result in isolates being falsely ascribed to clade II. Recently, Scheule et al. have developed a Ib-specific assay (dD14-16) targeting the flanking regions of the C3 L assay target (Schuele et al. 2024). This assay will not identify clade Ia strains, and hence either a triple test approach or sequencing is currently required to identify isolates to clade level. The C3 L gene codes for a complement control protein, and a homologue is also present in the variola (smallpox) virus, coding for the smallpox inhibitor of complement enzymes. These proteins are associated with inhibition of host complement activation pathways, which may contribute to virulence. The truncation of C3 L in clade Ib may therefore be a contributing factor to the apparently lower case fatality rate seen with this lineage, as demonstrated in animal experiments (Hudson et al. 2012).

Historically, electron microscopy has been used for direct detection of orthopoxviruses in clinical specimens. However, it cannot readily distinguish between species and is not recommended for routine use (World Health Organization 2024 (WHO)). Similarly, specific antibody detection may be used for surveillance but should not be used for primary diagnosis. Cross-reactivity from other orthopoxvirus exposure and/or vaccination is anticipated, and molecular methods are recommended by WHO as the primary diagnostic modality (Otter et al. 2023).

There is considerable interest in point-of-care (POC) testing for MPXV to improve access to testing; however, they have shown limited promise to date. A list of tests on the commercial market is maintained by FIND (2024). Two POC tests detecting MPXV nucleic acid have received FDA Emergency Use Authorization but have been validated for clade IIb only (World Health Organization (WHO), FDA 2023, U.S. Food and Drug Administration 2024). Commercial antigen POCs have also been produced and assessed, but details have not been published in peer-reviewed journals (Ishara-Nshombo et al. 2024). Similarly, in-house POC antigen assays exist but have yet to be formally evaluated and published (Laidlaw et al. 2024). Commercial antibody-based POC tests have also been assessed, with poor performance demonstrated (Jones et al. 2024).

## Immunology

Knowledge on MPXV immunology has largely been driven by that gained from utilizing smallpox vaccination during the eradication campaign in 1980, based primarily on the assumption of similarities between the highly similar variola (VARV), vaccinia (VACV), and MPXV viruses. The vast majority of MPXV cases from the Clade IIb outbreak were born after 1980 and the cessation of routine smallpox vaccination, driving the hypothesis that populations are once again susceptible to poxvirus infections due to lack of smallpox vaccine-driven immunity (Agrati et al. 2023).

As orthopoxviruses, both MPXV and VARV have a high degree of homology. The genome of MPXV is estimated to be ∼96%–98% similar to that of VARV, with key differences in virulence and host-range-related genes (Shchelkunov et al. 2001, Giorgi et al. 2024). Due to this high homology, immunity against MPXV is similar to that of VARV and vaccinia virus (VACV). Previously, it has been suggested that B cell-induced immunity (e.g. binding/neutralizing antibodies) drives protection from disease, whilst T cell and cellular immunity is primarily associated with control of infections (Xu et al. 2004, Jing et al. 2005), of which epitopes have been found to be conserved across members of the *Orthopoxvirus* genus (Jing et al. 2005, Grifoni et al. 2022).

The innate immune response is still poorly under-researched for all orthopoxviruses, including MPXV, with the majority of research focussed on VACV. However, recent work has demonstrated that MPXV primarily infects monocytes, which subsequently lead to dissemination of the virus with specific infection of CD4+ T cells and cycling NK cells (Bosquillon de Jarcy et al. 2024), supporting previous work undertaken on VACV (Sánchez-Puig et al. 2004). Upon infection of a host cell with an *Orthopoxvirus*, the DNA genome is released, and replication occurs directly within the host cell cytosol, in which host cytosolic DNA sensing is blocked, utilizing viral nuclease proteins such as VACV B2 and E5, often termed ‘poxins’. Similarly, IFN signalling is disrupted, and neighbouring NK cell responses can be partially inhibited through viral decoy cytokine receptors (Hernaez and Alcamí 2024). Whilst the majority of work has focussed on innate immunity to *Orthopoxvirus* infection, baseline inflammatory responses have been shown to significantly affect seroconversion to MVA-based vaccinations (Hernaez and Alcamí 2024). Further work is needed to elucidate differences in innate immunity from infection across both MPXV and/or vaccination in addition to comparison against other related members of the *Orthopox virus* genus.

The study of the adaptive immune response to both MPXV infections has similarly been based primarily on research gained from understanding VACV and associated vaccination.

A vital role in clearing *Orthopoxvirus* infections is driven by T cells, with MPXV-infected cells that can be directly killed by CD8+ cytotoxic T cells upon peptide presentation. It has been shown that during acute MPXV infection, effector CD4+ and CD8+ T cells are expanded, with 90% of individuals developing a poxvirus-specific Th1 response post-recovery, similar to the response seen with vaccination (86). A similar observation was observed in HIV-positive individuals, whereby robust CD4+ and CD*8+ T cells were detected against OPXV peptides (95). However, MPXV (and other OPXV) also harbour escape mechanisms from T cell-mediated clearance (96). T cell memory is robustly induced post-MPXV and post-vaccination, with cellular immunity detected in individuals multiple decades post-vaccination (86, 97) and cross-recognition across diverse OPXV due to high homology in activating peptides (95).

However, it has been shown from animal model studies that, in the absence of antibodies, T cells were necessary for survival and recovery in VACV challenge. Conversely, protection from disease was largely driven by humoral immunity, demonstrating that poxvirus immunity is a complex interplay between both humoral and cell-mediated immunity for which no known correlate of protection is currently known (96, 98).

Antibody-induced immunity against orthopoxviruses is primarily in the form of neutralizing antibodies; however, as typical with other infections, IgM antibodies are induced first with subsequent IgG-specific seroconversion (99). Smallpox/MVA-based vaccination induces similar immune responses, which have been detected as far out as 50 years post-vaccination (97, 100).

For neutralizing antibodies, the mechanism of protection from disease has been hypothesized to be through preventing viral entry and uncoating, with higher neutralizing titres correlated with higher protection (Belyakov et al. 2003, Zaeck et al. 2023, Selverian et al. 2024). However, non-neutralizing-binding antibodies can also contribute to immunity through Fc-dependent mechanisms, including complement activation (Cohen et al. 2011).

Both MPXV infection and VACV vaccination induce antibodies to a broad range of antigens; however, orthopoxviruses exist in two forms: an intracellular mature virion (IMV) primarily dominant during infection and an extracellular enveloped virus (EEV) associated with intra-host transmission. Each of these has distinct antigenic profiles, with differing proteins expressed and displayed on the surface of the virion. Approximately 25 proteins are expressed in the IMV form, whilst an additional 6 proteins are observed in the EEV form (Bengali et al. 2012, Moss 2012).

Previous work has demonstrated the difference in antigen recognition between IMV- and EEV-specific proteins (Townsend et al. 2013, Otter et al. 2023). Within these different forms, a range of immunodominant antigens are displayed on the virion surface, some of which are the target of neutralizing antibodies, whilst others provide the virion with defences against host immune responses. Immunity against a number of these immunodominant antigens is induced both by infection and MVA-based/VACV vaccination (Otter et al. 2023). However, MPXV infection and vaccination induce broadly similar immune responses against immunodominant antigens due to the high sequence homology in genes and proteins across OPXV (Otter et al. 2023). Within the IMV form, this includes surface proteins L1 (MPXV homologue M1), A27 (MPXV A29), H3, and D8 (MPXV E8), whilst in the EEV form, additional immunodominant outer membrane proteins are present, including A33 (MPXV A35) and B5 (MPXV B6) (Otter et al. 2023).

Within these immunodominant antigens, antigen reactivity has been found to be specific between vaccination and infection, with the MPXV A27 identified as a serological marker of MPXV infection (due to a gene truncation within the IMVANEX strain of MVA), whilst MPXV M1 (VACV L1) was found to be a marker of IMVANEX vaccination (Giorgi et al. 2024).

However, whilst much of the immunology work to date has focussed on VACV/smallpox vaccination, and clade IIb immunology, the genome similarity across MPXV clades suggests that whilst mutations occur between clades and sub-clades, genome similarity remains >99%, suggesting that MPXV clade IIb antigens can equally be used in detecting immune responses to the majority of MPXV clades, whereby very few mutations have occurred (Grifoni et al. 2022, Giorgi et al. 2024). However, no immunology work has currently been done that demonstrates cross-protection between MPXV clades.

## Vaccines and effectiveness

The high degree of virus homology and similarity in immune response has been the foundation of the use of smallpox vaccination as a control measure for mpox, particularly for the 2022 PHEIC. Current mpox programmes utilize third-generation smallpox vaccines, of which two are licensed for mpox prevention. These are LC16m8 (‘LC16’ or ‘KMB’), an attenuated, minimally replicating strain of vaccinia virus developed in Japan, and the modified Ankara (MVA-BN, Imvamune/Imvanex/JYNNEOS), which is a live, replication-deficient virus (Eslami et al. 2024, UKHSA 2025). Unlike first- and second-generation smallpox vaccines, MVA-BN does not require scarification and does not replicate at the site of inoculation, so it does not present a risk of auto-inoculation or inadvertent transmission, allowing its use in immunocompromised individuals.

The MVA-BN vaccine is licensed for use for mpox prevention in Canada, the USA, EU, the UK, and Switzerland. Analysis from the 2022 clade IIb PHEIC indicates efficacy in the prevention of mpox disease (Bertran et al. 2023). In England, the estimated vaccine effectiveness against symptomatic mpox at least 14 days after a single dose was 78% (95% CI 54–89) in an at-risk population, with a delayed second dose providing more durable protection (Berry et al. 2024).

There are no data for the effectiveness of MVA-BN for clade I MPXV, but there is no virological reason to infer lesser efficacy. Previous studies with second-generation vaccines in the DRC, and T-cell epitope mapping between clade I and clade IIb, suggest that there should be cross-protection (Ophinni et al. 2022, Ghazy et al. 2023).

People aged over 50 who were vaccinated in childhood as part of smallpox eradication measures may experience milder disease if infected with mpox (Eustaquio et al. 2023, Sanz-Muñoz et al. 2023).

## Treatment

Tecovirimat was approved by the FDA in 2018 for treatment of variola virus and in 2022 was brought into use for treatment of mpox, under the inference that it would have similar antiviral activity. Tecovirimat acts on the F13 L gene, which encodes a protein required for extracellular virus particle formation (Yang et al. 2005).

In spite of observational studies suggesting efficacy (Braddick and Singh 2024), a randomized, double-blind, placebo-controlled trial for treatment of clade I mpox (PALM007) did not show any significant improvement in resolution of skin lesions or clearance from various sample sites for tecovirimat vs. placebo (Tecovirimat for Clade I MPXV Infection in the Democratic Republic of Congo 2025). In part, this may be related to sub-optimal blood and tissue concentrations, but the conclusion was that for clade I at least, it is of limited clinical benefit at the currently adjudicated safe dose. Similar trials are ongoing for clade IIb, but may show comparable (and disappointing) results (clinicaltrials.gov. Study of Tecovirimat for Human Mpox Virus (STOMP), clinicaltrials.gov. Assessment of the Efficacy and Safety of Tecovirimat in Patients with Monkeypox Virus Disease (UNITY), clinicaltrials.gov. European Trial into Mpox Infection (EPOXI)). *In vitro* resistance to tecovirimat has already emerged, linked to mutations in the F13 L gene (Chenchula et al. 2025, Vernuccio et al. 2025).

Cidofovir is a viral DNA polymerase inhibitor, which shows activity vs. MPXV in animal models, but its use is limited by nephrotoxicity, hepatotoxicity, and requirement for IV administration. There is some evidence for *in vivo* efficacy, but numbers are small. Brincidofovir is a prodrug of cidofovir, which shows similar experimental activity against orthopoxviruses, but again, data on clinical efficacy are limited (Braddick and Singh 2024). Several other drugs are under evaluation (Hajjo et al. 2025), including topical treatments such as cantharidin, which has shown activity against molluscum, and trifluridine, which has been used for ocular disease (Guzman et al. 2018, Cinatl et al. 2024).

A further treatment strategy is the use of human vaccinia immune globulin (VIGIV), derived from individuals vaccinated against smallpox. VIGIV is FDA-approved for complications of vaccinia virus but, under some circumstances, may be considered for the management of related orthopoxviruses (e.g. development of sight-threatening ocular complications). However, there is minimal evidence for efficacy.

## Conclusion

Whilst mpox has been recognized as a potentially severe human disease for more than half a century, there is much still to be learned regarding fundamental factors such transmission, human infection dynamics, immune response, and infection prevention and control measures, and, as the last few years have demonstrated, emerging strains of MPXV are behaving differently from the classic, smallpox-like disease initially described in the 1970s and 1980s. This viral evolution requires a re-evaluation of established orthopox orthodoxy, particularly in understanding virological differences between the clades and sub-clades, which may influence virulence, transmission, and host preference, with the attendant implications for disease control.
